# Physiology of nitrogen: A life or death matter

**DOI:** 10.1113/EP092946

**Published:** 2025-06-20

**Authors:** Damian M. Bailey, Vaughan G. Macefield, David C. Poole

**Affiliations:** ^1^ Neurovascular Research Laboratory, Faculty of Life Sciences and Education University of South Wales Pontypridd UK; ^2^ Department of Neuroscience, School of Translational Medicine Monash University Melbourne Victoria Australia; ^3^ Department of Kinesiology Kansas State University Manhattan Kansas USA; ^4^ Department of Anatomy & Physiology Kansas State University Manhattan Kansas USA

**Keywords:** asphyxiation, assisted suicide, execution, inert, nitrogen gas

## Abstract

With each breath, four out of every five molecules we inspire are nitrogen (N_2_), since this gas constitutes ∼80% of the atmospheric air that surrounds us. Despite its abundance and unlike molecular oxygen, N_2_ has traditionally held less appeal among physiologists given its lack of reactivity and corresponding inability to support combustion or life, rendering it metabolically nugatory. The controversial application of N_2_ asphyxiation for the inhumane purposes of human execution of convicted criminals and assisted suicide of a terminally ill patient has thrust this important gas into the scientific and public spotlight, sparking widespread condemnation. In the current review, we take an opportunity to explore the molecular bases and clinical consequences linked to the Janus‐faced physiology of N_2_ to better explain its life‐and‐death qualities. We highlight the complex history that led to its discovery and the physio‐geochemical evolution of Earth's uniquely N_2_‐rich atmosphere, including intimate links with oxygen (O_2_), another life‐and‐death homonuclear diatomic gas that preceded aerobic respiration and the emergence of complex multicellular life. Diving deep into N_2_’s quantum state, we expose its unique physiochemical properties to better understand why this gas is metabolically inert and physiologically deadly when in excess and especially to the exclusion of O_2_. We apply this integrated physiological knowledge to further inform the controversial public debate and directly challenge the misconceived notion that N_2_ gas asphyxiation offers a quick, indolent and dignified death for the inhumane purposes of human execution and assisted suicide.

## Abstract (highlights)

- **What is the topic of this review?**
  The Janus‐faced physiology of life‐giving and life‐taking nitrogen, from its discovery, unique physio‐chemical properties that define ‘inertness’, to its misguided application for human execution of convicted criminals and assisted suicide in the terminally ill.
- **What advances does it highlight?**
  We address critically the inhumane and ultimately flawed practice of nitrogen gas asphyxiation for human execution and assisted suicide, challenging the widely held misperception that this offers a quick, indolent and dignified death, laid bare by physiological facts as opposed to misinformed political fiction.

‘Neither will I administer a poison to anybody when asked to do so, nor will I suggest such a course.’ Hippocrates, 460–370 BCE.

## 1 INTRODUCTION

With every resting breath, we inspire a staggering ∼1.6 × 10^21^ molecules of dinitrogen gas (N_2_), comprising ∼78% of the ∼2.0 × 10^21^ (sextillion) molecules of atmospheric air that surrounds us (Box 1). To put the magnitude of this number into clearer context, convert the 4.54 (± 0.05) billion years that reflects the geological age of Earth into seconds: you will still fall short by more than four orders of magnitude! Yet, remarkably, of the sextillion molecules of N_2_ that enter our lungs with each and every breath, that same sextillion exits, untouched: and all because N_2_ is metabolically inert (Poole & Whipp, 1988).

**BOX 1:** Every breath you take.

**Calculating how much N_2_ we inspire with each breath**: Assuming 0.5 L tidal volume and 37°C body temperature and application of Ideal Gas Law *PV* = *nRT* $n=\frac{PV}{RT}$ $n=\frac{1\;(\mathrm{atm})\;\times \;0.5\;(L)}{0.08206\;(L/\mathrm{atm}/\mathrm{mol}/K)\;\times \;310.15\;(K)}$ = 0.0196 mol Number of molecules of air = *n*(0.0196) x Avogadro's number (6.022 × 10^23^) = 1.96 × 10^21^ Number of molecules of N_2_ = *n*(0.0196) × mole fraction (0.781) × Avogadro's number (6.022 × 10^23^) = 1.53 × 10^21^

Abbreviations: *n*, number of moles; *P*, pressure; *R*, gas constant; *T*, temperature in Kelvins at 1 atmosphere (atm) and (body) temperature of 37°C; *V*, volume.

This lack of reactivity and corresponding inability to support combustion or life, unlike molecular oxygen (O_2_), is perhaps why chemists and physiologists alike have tended to overlook a gas originally labelled *mephitic* (noxious or poisonous) or *azotic* (lifeless) during the multifaceted course of its discovery in 1772 by Daniel Rutherford (Rutherford, 1772). Joseph Priestley (1733–1804) subsequently isolated ‘dephlogisticated air’ some 2 years later in 1774 and it proved to be a far more fashionable discovery, with O_2_ quickly taking centre stage given its ‘magical’ ability to reignite burning embers and extending the survival of mice in a closed container (Priestley, 1776). As a consequence, N_2_ has historically been considered the ‘stepchild among respiratory gases’ (Rahn, 1961).

Modern times have seen a resurgence of interest in the physiology of N_2_, especially when it is encountered at elevated partial pressures (hyperbaria) and given its relevance to our understanding of nitrogen narcosis and decompression sickness among divers and astronauts (notwithstanding its indisputable importance for the biosphere and cellular life). Recent application of N_2_ gas asphyxiation (from the Greek α, meaning ‘without’, and σφυγμóς (sphygmos), meaning ‘pulse or heartbeat’), which effectively displaces or replaces O_2_ for the inhumane purposes of human execution of convicted criminals in the USA and assisted suicide of a terminally ill patient in Switzerland, has thrust this important gas into the scientific and public spotlight, albeit on account of its use for nefarious purposes.

There are ‘other’ experimental approaches to acutely induce systemic anoxia, notably the following. (1) Environmental (inspiratory hypoxia at extreme high‐altitude). (2) Chemical, including mitochondrial electron transport chain inhibitors such as rotenone, antimycin A or oligomycin; cobalt chloride, which serves to mimic hypoxia by stabilizing hypoxia‐inducible factor 1‐alpha (HIF‐1 α); desferrioxamine, an iron chelator that serves to stabilize HIF; sodium azide, which inhibits cytochrome *c* oxidase, mimicking hypoxia at the cellular level; and poisoning with carbon monoxide, which forms carboxyhaemoglobin and also binds to cytochrome *c* oxidase, reducing the oxygen‐carrying capacity of blood. (3) Mechanical obstruction, which serves to induce cerebral ischaemia, which includes inflation of a specialized cervical pressure cuff described in the (infamous) Red Wing studies (Rossen et al., 1943) (see ‘Physiology of death by N_2_ asphyxiation: facts over fallacy’). And (4) Genetic manipulation, which includes knockdown of HIF regulators and overexpression of HIFs to induce hypoxia‐mimicking gene expression. Although not all approaches have been interrogated in the human model to date for obvious ethical reasons, their contribution to the death process is likely to be very different.

In this contemporary review, we will specifically focus on the inert gas N_2_, which also happens to be our bulk atmospheric and oceanic gas in the presence of which all life has evolved. We take an opportunity to shed light on the molecular structure of N_2_ to better appreciate its unique physiochemical properties. We explore the complex history underlying its discovery, the evolution of Earth's N_2_‐rich atmosphere and, importantly for physiologists, N_2_’s unique physiochemical properties that define ‘inertness’. Finally, we address critically the inhumane and ultimately flawed practice of N_2_ gas asphyxiation for human execution and assisted suicide, challenging the widely held misperception that this offers a quick, indolent and dignified death, laid bare by physiological facts as opposed to speculative and misinformed political fiction. As clearly stated previously in Macefield (2024) and Poole & Bailey (2024), we vehemently oppose the death penalty, given that it is inconsistent with the fundamental right to life, and support the UN human rights office opposition to N_2_ anoxia as a method of execution (Shamdasani, 2024).

## 2 DISCOVERY OF N_2_: FROM MALIGNANT AIR TO NITROGÈNE

**FIGURE 1:**
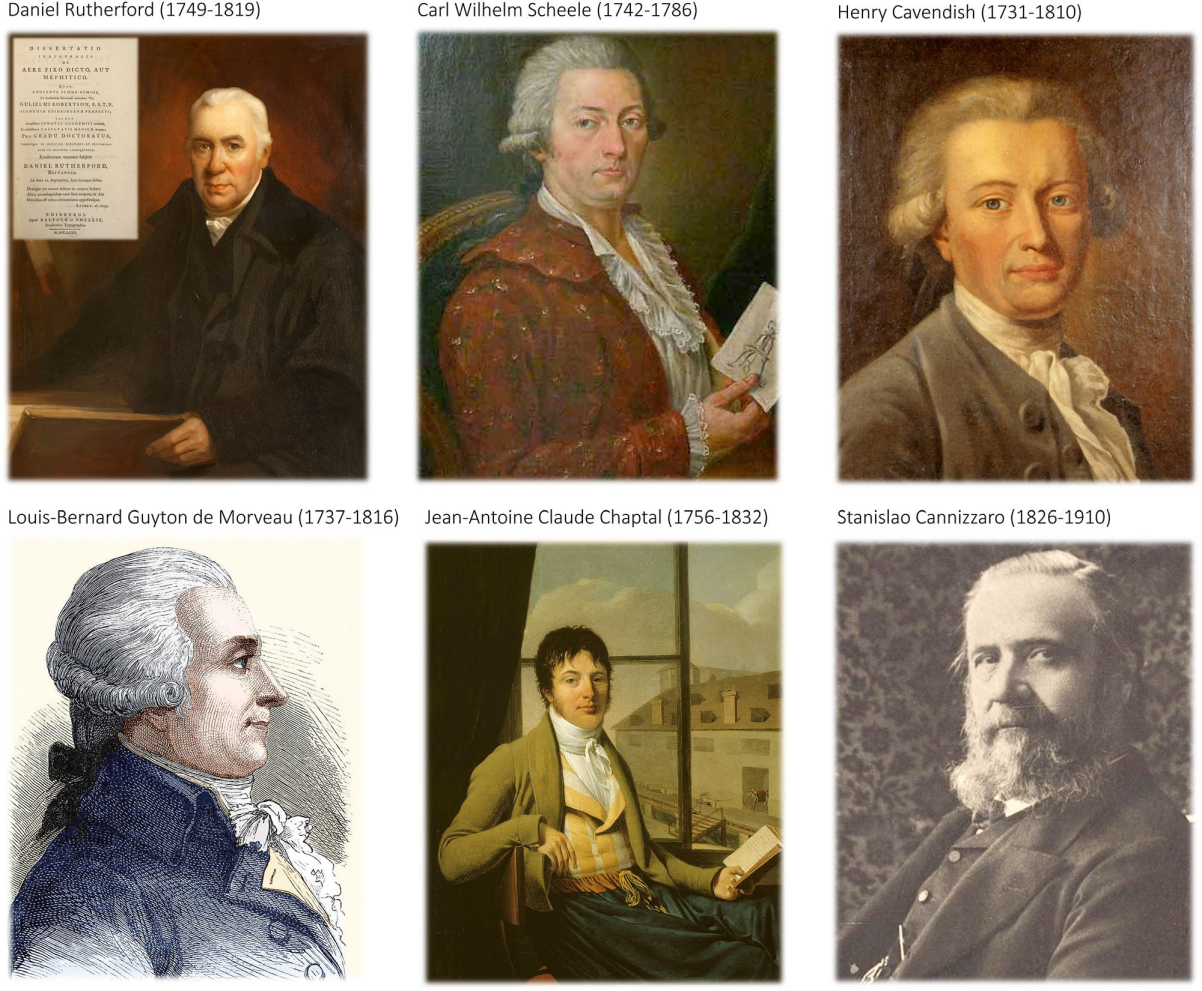
Scientists who contributed towards the discovery and characterisation of nitrogen. Image credits: Royal College of Physicians of Edinburgh (Daniel Rutherford); Public domain, Wikidata or Wikimedia Commons (Carl Wilhelm Scheele, Henry Cavendish, Louis‐Bernard Guyton de Morveau, Jean‐Antoine Claude Chaptal, Stanislao Cannizzaro).

The discovery of N_2_ is generally credited to the Scottish scientist Daniel Rutherford (Figure 1) in 1772 (Lavoisier, 1790); he studied medicine at the University of Edinburgh under William Cullen (1710–1790) and Joseph Black (1728–1799). Extending Black's seminal works and original discovery of ‘fixed’ or ‘mephitic’ air (carbon dioxide, CO_2_), Rutherford ultimately made his mark by establishing a distinction between carbonic acid gas and ‘noxious’ or ‘malignant’ air, later to become known as N_2_. Other scientific luminaries were also hard at work, notably Carl Wilhelm Scheele – who many consider the co‐discoverer of N_2_, referring to ‘spoiled’ air – and Henry Cavendish (Figure 1), who considered the gas to be the consequence of the ‘destruction of common air’ (Priestley, 1772). However, taking the same approach that Joseph Priestley adopted some 2 years later with O_2_, Rutherford had the foresight to be the first to formally publish his discovery of this ‘new’ air in his 1772 MD dissertation entitled ‘Inaugural dissertation on the air called fixed or mephitic’ (Dobbins, 1935; Rutherford, 1772) (Figure 1).

In his thesis (Dobbins, 1935; Rutherford, 1772), Rutherford described the destruction of ‘ordinary’ air (i.e., he removed the dephlogisticated air or O_2_ based on Priestley's soon to be antiquated ‘phlogiston theory’ – originally promulgated by Georg Ernst Stahl (1659–1734) (West, 2014) – by burning charcoal or a candle, or through addition of a living (respiring) mouse. He removed CO_2_ with caustic lixivium (a solution containing alkaline salts), a procedure originally developed by Black, concluding that ‘malignant’ air must be'…atmospheric air saturated with phlogiston’ given that it ‘…cannot be converted into mephitic air by combustion’. He did not name his air, instead speculating that it was ‘…pure phlogiston united to common air’ seeming to ‘…form another species of air’.

Rutherford was both fascinated and puzzled with his new air, quick to highlight that when ‘all mephitic air had been removed by caustic lixivium, what remains does not become in any way more wholesome’, later noting that air ‘which has been blown through ignited coals, and then purified from all mephitic air, is nevertheless still found to be malignant and quite similar to that which is spoiled by respiration.’ Although he observed that the ‘malignancy’ (since mice exposed to ‘phlogisted air’ failed to survive) induced by respiration, combustion or calcining was a separate phenomenon from the ‘mephitic’ nature of fixed air, he never fully resolved the differences between the two diatomic gases.

The phlogisticated or malignant air's modern name took almost two decades to formalise, changing from ‘mofette’ (gas escaping from a volcanic vent, suggested by Antoine‐Laurent Lavoisier), to ‘azote’, from the Greek word, άζωτικός (azotikos), which translates into ‘without life’ (by Louis‐Bernard Guyton de Morveau, Figure 1), and ‘azotic gas’ (Henry Cavendish). We had to wait until the French chemist Jean‐Antoine Claude Chaptel (Figure 1) ultimately named the gas ‘nitrogène’ in 1790, a reference to nitre (potassium nitrate), which was known to contain the element N (Partington, 1962). Finally, it was not until 1858 that Stanislao Cannizzaro (1826–1910) (Figure 1) finally elucidated the true nature of the gas, which owing to its covalent bonds occurs naturally as diatomic N_2_ (Cannizzaro, 1858).

## 3 ATMOSPHERIC N_2_: PLANETARY EVOLUTION

**FIGURE 2:**
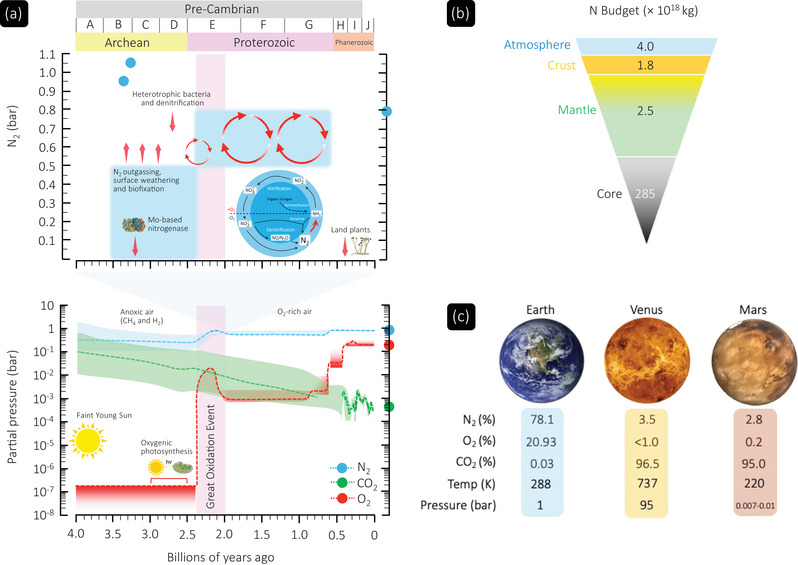
Planetary evolution of atmospheric nitrogen including Earth's present‐day budget and reservoirs. (a) Lower and upper panels, modified from Catling & Zahnle (2020). Lower panel illustrates post‐Archean evolution of Earth's atmospheric nitrogen (N_2_), oxygen (O_2_) and carbon dioxide (CO_2_). Shading reflects uncertainties relating to gas concentrations. Upper panel provides an expanded view of the N_2_ timeline, defined by a wide range of partial pressures (blue shading) due to limited proxy data. Blue circles reflect (upper limit) present‐day data points (Avice et al., 2018; Berner, 2006; Marty et al., 2013). Given its stability, dinitrogen gas (N_2_) needs to undergo multiple transformations including fixation, nitrification, denitrification, anammox, and ammonification before it becomes biologically available (highlighted). A, Eoarchean; B, Paleoarchean; C, Mesoarchean; D, Neoarchean; E, Paleoproterozoic; F, Mesoproterozoic; G, Neoproterozoic; H, Palaeozoic; I, Mesozoic; J, Cenozoic. (b) Figure based on data from Li (2024). (c). Atmospheric composition of sibling planets based on data from Franz et al. (2017), Oyama et al. (1980) and Prinn & Fegley Jr (1987). CH_4_, methane; H_2_, hydrogen. Timeline of molybdenum (Mo)‐based nitrogenase from Stueken et al. (2015).

Despite nitrogen comprising 78.1% by volume of the atmosphere (78.3 atom% or 75.5 wt%) and being of fundamental importance for all known life, our understanding of its geochemistry encompassing delivery, speciation, isotopic evolution and cycling between Earth's crust, mantle and atmosphere still remains incomplete (Wordsworth, 2016). The general view is that before the Great Oxidation Event (GOE, ∼2.4–2.1 billion years ago [Gya]), the anoxic Archean (spanning 4–2.5 Gya) atmosphere was dominated by the non‐condensable greenhouse gases, CO_2_ and methane, which countered the Faint Young Sun's ∼25–30% lower solar luminosity ∼4 Gya (Catling & Zahnle, 2020) (Figure 2a).

Although precise estimates are highly variable given uncertainties relating to the geological N cycle, N_2_ was the most abundant bulk gas, present at similar if not potentially lower paleopressures (*P*) than present‐day levels (Figure 2a). The rise in N_2_ likely shadowed the rapid elevation in O_2_ levels during the GOE subsequent to oxidative weathering and denitrification. The emergence of biological (biotic) nitrogen fixation through the advent of a molybdenum‐based nitrogenase (∼3.2 Gya) (Stueken et al., 2015) would have markedly reduced the $P_{N_{2}}$ of the Archean atmosphere, with levels changing little over the Phanerozoic Eon (Berner, 2006) (Figure 2a).

The present‐day atmosphere continues to be dominated by N_2_, which remains one of Earth's largest N reservoirs. At an estimated 4 × 10^18 ^kg (Figure 2b), atmospheric N_2_ is a major player in our climate because it causes pressure broadening of the absorption lines of greenhouse gases (Goldblatt et al., 2009). Indeed, since pre‐industrial times, anthropogenic fossil fuel combustion and synthetic fertilizer application through industrial fixation of N_2_ into ammonia (NH_3_) via the Haber–Bosch process (N_2_ + 3H_2_ ⇌ 2NH_3_, $\Delta H_{298K}^{o}$ = −92.28 kJ/mol) (Haber, 1920) have had more of an impact on the nitrogen cycle than any other major biogeochemical cycle, substantially enhancing ‘fixed’ (reactive) nitrogen and contributing to widespread eutrophication and air pollution (Gong et al., 2024). This contrasts starkly with the atmospheric composition of our planetary siblings, Venus and Mars, which share atmospheres dominated by CO_2_ (Figure 2c) (Prinn & Fegley Jr, 1987). The elevated atmospheric N_2_ content on Venus (which is related to the much higher atmospheric pressure) is explained abiotically, whereby O_2_ liberated from H_2_O photolysis and subsequent hydrogen loss to space oxidises the mantle, thus accelerating N_2_ outgassing. The lower atmospheric N_2_ content on Mars is mostly attributable to increased escape to space and storage in the mantle (Wordsworth, 2016).

## 4 THE JANUS FACE OF N_2_: INSIGHTS INTO INERTNESS

Before considering the physiology of N_2_, it is important to fully understand its physical chemistry. Indeed, it is well established that the integration of chemical principles can provide unique mechanistic insight into complex physiological processes, from cellular functions to systemic regulation (Day, 1861).

While molecular N_2_ is inert with an atmospheric lifetime of ∼1 Gya (Berner, 2006), O_2_ is a highly reactive di‐radical requiring continuous generation via oxygenic photosynthesis, a unique development that preceded aerobic respiration and subsequent emergence of complex multicellular life (Bailey, 2019b). Nitrogen nestles between carbon and oxygen, the seventh element in Group 15 of the periodic table, befittingly known as the pnictogens, derived from the Greek word ‘pnigein’ – ‘to choke’ or ‘stifle’. While physiologists are familiar with the essential importance of nitrogen as a fundamental constituent of nucleic acids and proteins, notwithstanding myriad commercial and biomedical applications (Farhi, 1964; Tarselli, 2012), what defines its ‘inertness’ and corresponding deadly asphyxiant properties is arguably less well understood, demanding a closer look at this molecule's unique physio‐chemical properties.

**FIGURE 3:**
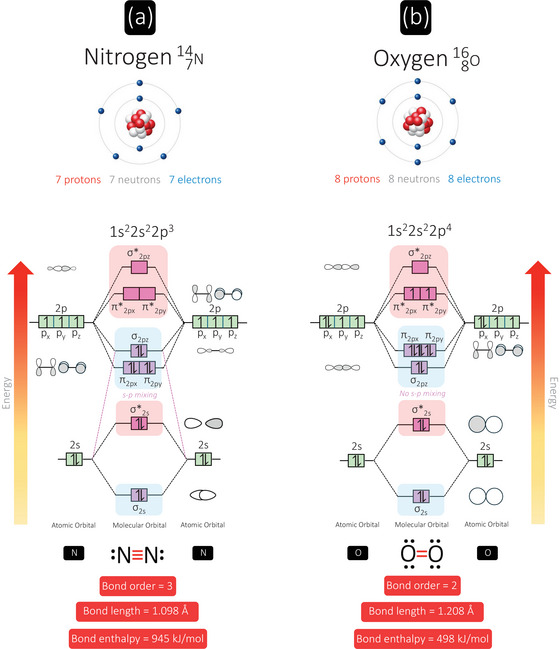
Molecular orbital (MO) energy level diagrams for nitrogen and oxygen: ‘foul’ and ‘fire’ air. Each line represents a MO and the arrows represent electrons, the direction of which indicates their spin quantum number. Bonding MOs (↓energy/↑stability) are shaded in blue and antibonding orbitals (↑energy/↓stability) are shaded in red for each molecule. Bond order is calculated as: (bonding electrons − antibonding electrons)/2. Note that the 1s orbitals are not illustrated given that they have the same configuration as 2s orbitals, and that the energies of the atomic orbitals are not drawn to scale. Bond lengths taken from the National Institute of Standards and Technology database (NIST, 2023). Bond disassociation enthalpies taken from Luo (2003) and cited in Das & Arunan (2019).

This can be best understood through application of molecular orbital (MO) theory, a chemical framework originally developed by Hund (Hund, 1928, 1931) and Mulliken (Mulliken, 1928a, 1928b, 1928c, 1932), which physiologists can employ to fully appreciate how atoms combine to form molecules and how electrons are distributed within them in accordance with Hund's rule (Hund, 1925) and the Aufbau (Bohr, 1921) and Pauli Exclusion Principles (Pauli, 1925). The atomic configurations and molecular electronic structures for two of the period 2 homonuclear diatomic gases, N_2_ and O_2_, are compared and contrasted in Figure 3a,b, united by their Janus‐faced physiology.

In the ground‐state electron configuration of the N_2_ molecule, each N atom contributes five valence electrons (two in 2s and three in 2p orbitals), resulting in a total of 10 electrons that need to be distributed among the MOs (Figure 3a). Two electrons pair (⥮), occupying and filling the σ_2s_ (bonding) orbital, while the next two occupy and fill the σ*_2s_ (anti‐bonding) orbital, resulting in no net bonding. The remaining six electrons fill the π_2px_, π_2py_ and σ_2pz_ orbitals, with no ‘destabilising’ electrons occupying the antibonding orbitals (i.e., they are vacant). Thus, the predicted bond order is calculated as: (bonding electrons – antibonding electrons)/2 = (8 − 2)/2 = 3, highlighting that a triple covalent bond (one σ and two π bonds) exists between two N atoms (also predicted by the more simplistic Lewis structure (Lewis, 1916), bottom of Figure 3a).

Thus, N_2_ is the most strongly bound diatom, agreeing with its very short internuclear bond distance (1.098 Å) and high disassociation enthalpy (945 kJ mol^−1^ at 298 K), the highest for all seven homonuclear diatomic molecules in the periodic table. Furthermore, since N_2_ does not house any unpaired electrons, it is diamagnetic. It is precisely the strength and non‐polarity of this triple bond that underlies N_2_’s exceptional stability and lack of reactivity or ‘inertness’. This also helps explain why biological nitrogen fixation (Figure 2a) is constrained to only a select group of prokaryotes given that it is such a bioenergetically demanding process, requiring 8 electrons and ∼16 molecules of ATP per molecule of N_2_ fixed (N_2_ + 8H^+^ + 8e^−^ → 2NH_3_ + H_2_). The industrial fixation of N_2_ via the aforementioned Haber–Bosch process (Haber, 1920) utilizes iron catalysts at high pressures and temperatures to overcome the activation energy required to break the formidable N≡N bond, supplementing the 3‐billion‐year‐old molecular innovations. However, while this process has been instrumental in feeding a growing global population, more than quadrupling the productivity of agricultural crops, chemical fertilizers and other anthropogenic sources of fixed nitrogen far exceed natural contributions, leading to unprecedented environmental degradation (Stein & Klotz, 2016).

Compare and contrast this with the ground‐state electron configuration of the reactive O_2_ molecule, which has one more electron from each atom and thus yields a total of 12 valence electrons (Figure 3b, see Bailey (2019b) for a comprehensive review including physiological implications). Two electrons fill each of the σ_2s_ and σ*_2s_ orbitals, two more fill the σ_2pz_ orbital and four fill the degenerate π_2px_ and π_2py_ orbitals. Note the difference in the position of the σ_pz_ orbital, which is lower in energy than the π_px_ and π_py_ orbitals compared to N_2_ due to s–p mixing in the latter (highlighted by purple stippled lines). According to Hund's rule, the last two electrons must be placed in separate π*_2px_ and π*_2py_ orbitals with the same quantum spin number (i.e., parallel spins), giving a net spin angular momentum of 1 (triplet state) with two unpaired electrons, which energetically ‘cancel out’ one of the π*_2p_ orbitals. Hence O_2_ qualifies as a di‐radical and it is paramagnetic (attracted to a magnetic field), a long‐standing puzzle that could not be predicted by other, arguably more inferior bonding models (see Lewis structure, bottom of Figure 3b). It is this paramagnetic quality of O_2_ that lends its utility to functional magnetic resonance imaging of the brain, in which changes in blood oxygen level dependent (BOLD) signal reflect changes in O_2_ uptake related to synaptic energetics (Logothetis, 2002). Thus, O_2_ has a predicted bond order of (8 − 4)/2 = 2, which corresponds to a double covalent bond (one σ and one π bond), which agrees with its (longer) bond length (1.208 Å), and (lower) disassociation enthalpy (498 kJ mol^−1^ at 298 K) compared to N_2_.

## 5 NEBULOUS NATURE OF N_2_: ASPHYXIATION FOR HUMAN EXECUTION AND ASSISTED SUICIDE

Unfortunately, in the USA, death penalty states – including Alabama, Mississippi, Nebraska and Oklahoma – have recently passed or are currently considering legislation to approve the use of N_2_ gas asphyxiation as an alternative method of human execution. This controversial approach in which the convicted criminal is forced to inhale supposedly pure N_2_ gas supplied into a mask has recently evolved in response to the limited availability of and practical challenges to drugs used for lethal injections (Wise, 2024). In 2024, the state of Alabama was the first to officially sanction the execution of three inmates (Kenneth Eugene Smith, the first to be asphyxiated on 25 January, Alan Eugene Miller on 26 September, and most recently, Carey Grayson on 21 November 2024).

Understandably, this stoked global outrage including a published statement from the UN Human Rights Office that the ‘novel and untested method… could amount to torture or other cruel, inhuman or degrading treatment or punishment under international human rights law’ (Shamdasani, 2024). Indeed, the American Veterinary Medical Association euthanasia guidelines states that nitrogen asphyxiation can be an acceptable method of euthanasia under certain conditions for pigs but not for other mammals, because it creates an ‘anoxic environment that is distressing for some species’ (Leary et al., 2020). We recently condemned this approach, basing our arguments on physiological observations from eyewitness testimonies in conjunction with extant literature and application of established physiological knowledge of the control of breathing and principles of cerebral bioenergetics, to directly oppose the misconceived perception that N_2_ asphyxiation causes the ‘swift, painless and humane’ death predicted by state authorities (Macefield, 2024; Poole & Bailey, 2024). These concerns also extend to the misappropriated deployment of N_2_ that evidence suggests is superseding helium as the (inert) gas of choice in plastic bag and other (non‐state imposed) asphyxial suicides given concerns that helium may be adulterated with air or O_2_ and therefore not be as effective (Byard, 2018). Other gases, including the potent anaesthetic hydrogen sulfide are also being ‘considered’, which may attract separate attention in light of its ability to further accelerate loss of consciousness (LOC) and cause death via distinct physiological mechanism(s), similar to that of cyanide toxicity (Ruder et al., 2015).

More recently, N_2_ gas asphyxiation has been applied within the context of assisted suicide following controversial inception of the futuristic ‘Sarco’ (an abbreviation of ‘Sarcophagus’) pod by its Australian inventor and euthanasia activist, Dr Philip Nitschke, whose medical licence was temporarily suspended in 2014. This remains a highly controversial and emotive moral issue, with clinical, legal, political, religious and ethical ramifications complicated by continuing demand for amendments to current legislations that have been discussed in detail (Picon‐Jaimes et al., 2022). Several foundational reviews provide diverse perspectives encompassing moral, legal and professional considerations of the ethical challenges surrounding assisted dying, reflecting fierce and unremitting debates in medical ethics (Campbell, 2019; Richards, 2025; Sinmyee et al., 2019). However, the current debate is as much about a physiological misunderstanding as it is an ethical one, underscoring the need for a more informed examination of N_2_ asphyxiation to ensure it aligns with sound physiological rationale and principles, ethical standards and constitutional protections.

**FIGURE 4:**
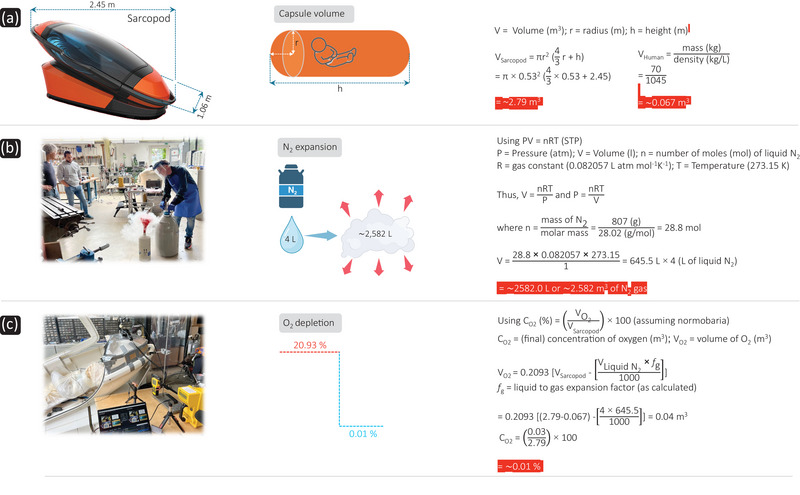
Nitrogen gas asphyxiation with the Sarco for assisted suicide: concepts, calculations and consequences. (a) Concept of the ‘Sarco’‐assisted suicide pod. Approximation of the ‘Sarco’ volume based on published dimensions taken from https://www.exitinternational.net/sarco/specs/. Human volume (simply space occupied assuming no gaseous exchange) is calculated for a 70 kg person who is 1.70 m in stature, with density predicted using the Brozek formula (density = 1.097 − 0.00046971 × mass + 0.00000056 × mass^2^ − 0.00012828 × stature). (b) Application of the ideal gas law to demonstrate (N_2_) liquid to gas volume expansion (assuming the interior of the pod remains normobaric with extant oxygen (O_2_) and carbon dioxide expelled via a one‐way valve system located at the rear of the Sarco as publicised, with little‐or‐no mixing, which if it occurred, would retain O_2_ in the pod: https://www.exitinternational.net/sarco/testing/). The documented volume of liquid N_2_ (4 L) will evolve ∼2582 L of inert N_2_ gas under standard temperature and pressure (STP) conditions, of ∼646 times its original volume (liquid to gas expansion factor). (c). Calculation demonstrating to what extent N_2_ gas will result in O_2_ depletion within 60 s to yield a near anoxic inspirate based on the volumetric assumptions outlined in (a) and assuming immediate and uniform introduction of the N_2_ gas under normobaria that washes out extant O_2_. All photos (a–c) from https://www.exitinternational.net/sarco/.

The ‘Sarco’ is a portable euthanasia device that can be moved to a panoramic location and consists of a 3D‐printed detachable capsule mounted on a stand containing a (4 L) canister of liquid N_2_ (Figure 4a). Once sealed inside the pod, the user is required to enter an activation code on a touchpad and answer automated questions about their identity and understanding of the process, before pressing a button to activate release of liquid N_2_, which displaces the extant O_2_ and CO_2_‐containing air, at the prevailing barometric pressure (normobaria), thereby inducing asphyxiation and death (see Figure 4b, c for calculations and consequences). An emergency exit button is also located inside the pod, providing a potential escape route if required. The biodegradable wood amalgam capsule can be detached allowing it to serve as the deceased individual's coffin; a one‐stop shop!

The Last Resort organisation deployed the Sarco for the first time in the assisted suicide of an unnamed American woman who ‘had been suffering for many years from a number of serious problems associated with severe immune compromise’ at a private forest retreat in the canton of Schaffhausen in northern Switzerland on 23 September 2024. While we do not oppose an individual's right to euthanasia per se, the means of providing assisted suicide is not so simple. Indeed, assisted suicide has been legalized in Switzerland assuming the person takes their own life with no ‘external assistance’ and those facilitating such are not doing so for ‘any self‐serving motive’, but this event triggered an ongoing criminal investigation (O'Dowd, 2024). The biased opinion of its proponents also incorrectly argues that the rapid reduction in O_2_, while maintaining low CO_2_ levels, will result in a peaceful, if not euphoric death. Herein, we take the opportunity to elaborate on our original critique (Macefield, 2024; Poole & Bailey, 2024) and provide more detailed insight into the integrative physiology of death by N_2_ asphyxiation.

## 6 PHYSIOLOGY OF DEATH BY N_2_ ASPHYXIATION: FACTS OVER FALLACY

**FIGURE 5:**
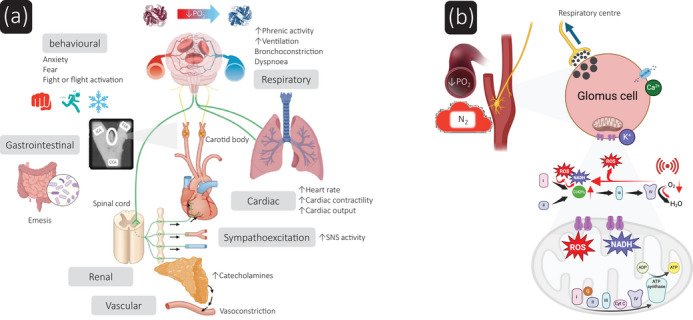
Integrated physiological responses and redox‐regulated oxygen‐sensing/transduction pathways evoked by the carotid bodies when breathing anoxic gas (100% nitrogen). (a) Location, innervation and activation of the carotid bodies underlying integrative organ systems responses. Note normal ovoid left carotid body (typically weighing ∼8 mg, roughly the same size as a grain of rice). CCA, common carotid artery; ECA, external carotid artery; ICA, internal carotid artery. Adapted from Khan et al. (1988). (b) Mitochondria‐to‐membrane signalling model of acute oxygen sensing by glomus cells highlighting substrates and functional interaction with redox‐regulated membrane ion channels. Figure adapted from Ortega‐Sáenz et al. (2020) and Fernandez‐Aguera et al. (2015). N_2_, nitrogen; PO_2_, partial pressure of oxygen; ROS, reactive oxygen species.

Nestled in the bifurcation of the common carotid arteries and innervated by the glossopharyngeal nerve (IX cranial nerve) lie the peripheral chemoreceptors or carotid bodies (CBs). The CBs continuously ‘taste’ the oxygen pressure in the arterial blood, which fluctuates over the course of each breath. Positioned just a few seconds downstream from the lungs, these organs constitute our body's sole sentinels that guard against arterial hypoxaemia, stimulating breathing when low O_2_ is detected. Described initially by Taube, a student of Albrecht von Haller, in 1743 (Taube, 1743) and called the ‘ganglion minutuum’ on account of their tiny size, the CBs weigh only a few milligrams (Figure 5a). However, although respiration is primarily determined by the level of CO_2_, their role in ventilatory control is essential, and was described initially by Jean‐Francois Heymans and his son, Corneille, in Ghent, Belgium: a discovery that won Corneille the Nobel Prize in 1938 (Heymans & Heymans, 1927; Kumar & Prabhakar, 2012).

Per unit mass, the CBs may be subject to the highest blood flow of any organ, yet they extract only ∼3% of their inflowing O_2_. This property helps facilitate their exquisite sensitivity and rapid response to a reduction in arterial *P*_O2_ (Black et al., 1971; Kumar & Prabhakar, 2012). Denervation of the CBs correspondingly abolishes the acute response to hypoxia, impairs adaptation to high altitude (Roeggla et al., 1995) and relieves the incapacitating dyspnoea in children with severe bronchial asthma (Timmers et al., 2003; Winter, 1973, 1979).

Signals of acute hypoxia are sensed by the CBs and transmitted via the glossopharyngeal nerve to the nucleus tractus solitarius (NTS) in the medulla (Figure 5a). A mitochondria‐to‐membrane signalling model of acute O_2_ sensing has been proposed, whereby activation of glomus cells by hypoxia ultimately depends on mitochondrial cytochrome *c* oxidase‐induced formation of NADH and reactive oxygen species, which modulate membrane ion channels to induce depolarization, Ca^2+^ influx and transmitter release (Ortega‐Sáenz et al., 2020) (Figure 5b). Direct excitatory projections from the NTS to the rostral ventrolateral medulla lead to increases in sympathetic vasoconstrictor drive to the muscle, splanchnic and renal vascular beds, reducing blood flow to these tissues and thereby increasing blood pressure and hence perfusion to the vital organs – the heart and brain (Zoccal et al., 2024) (Figure 5a). The spleen also contracts within a few seconds, liberating additional red blood cells and O_2_‐carrying capacity into the circulation (Pernett et al., 2021). Importantly, excitatory projections from the NTS run directly to the brainstem respiratory complex, including the preBötzinger and Bötzinger nuclei to elevate motor drive to the inspiratory and expiratory muscles, respectively (Zoccal et al., 2024). Moreover, these signals also generate perceptions of air hunger and dyspnoea, the former first described in 1892 (Haldane & Smith, 1892), and stimulate multiple other higher centres outside the brainstem. These centres include the insular cortex, which integrates perceptions relevant to homeostasis, including thirst, food hunger and pain, and limbic structures associated with fear and anxiety (Figure 5a).

In 1926 the physician Charles F. Hoover wrote of his clinical experience with air hunger (Hoover, 1926):

By air hunger is meant the subjective experience of air want. Dyspnoea, tachypnoea, hyperpnoea and cyanosis are attendant phenomena and can be evaluated by objective observation, but the feeling of air hunger is what alarms the patient and leads him to seek medical aid. The physician is called for a single reason and that is to relieve air hunger, a subjective symptom that is always alarming to the patient.

Thus, dyspnoea or air hunger are primary symptoms and drive a rapid elevation of sympathetic activity to the heart, blood vessels and adrenal glands (Figure 5a) that are so profoundly disturbing that they are responsible for people seeking medical care for their relief. Any increase in ventilation may be associated with dyspnoea, defined as an increased level of breathing discomfort, akin to breathlessness, shortness of breath or air hunger, but for any level of ventilation, hypoxic stimulation of hyperpnoea is especially ‘dyspnoea‐genic’ (Ward & Whipp, 1989). That the CBs generate dyspnoeic respiratory sensations is also supported by the fact that breath‐hold (BH) duration is shortened by reductions in inspired O_2_ prior to the voluntary apnoea, a response largely absent in CB‐resected patients; BH duration is greatly increased following CB resection (Davidson et al., 1974). Also consistent with a focal role for the CBs in dyspnoea is that inspired hyperoxia renders them quiescent, almost eliminating afferent traffic in the glossopharyngeal nerve, and reducing or abolishing dyspnoea (Chronos et al., 1988).

During BH, air hunger has been described as a primal sensation, signalling failure to sustain gas exchange, one of our most conserved homeostatic demands. Air hunger occurs when actual ventilation is perceived to be lower than the required ventilation, for example, to control arterial blood gases and maintain acid–base balance. Thus, it is believed that medullary respiratory centres project a copy of motor activity to the sensory cortex where it is compared to feedback from pulmonary stretch receptors and chest‐wall afferents signalling tidal lung inflation (Banzett et al., 2021). This is the basis for the concept of length–tension inappropriateness of the respiratory muscles that drives the powerful sensations of air hunger and dyspnoea and is especially prevalent in pulmonary disease patients (Bennett et al., 1963). Marked dyspnoea occurs at a lower threshold for increases in (alveolar) $P_{\mathrm{AC}O_{2}}$ (10 mmHg) than decreases in $P_{AO_{2}}$ (40–50 mmHg) and the perception of air hunger with hypoxia is similar to that experienced with normoxic hypercapnia, supporting the idea that it is the central drive to breathe that leads to the dyspnoea (Moosavi et al., 2003). Moreover, even in experimentally paralysed participants, the perceptions of extreme air hunger at the apnoeic breaking point occur at the same latency as that experienced during a voluntary apnoea, indicating that it is central command, rather than the increased work of the respiratory muscles, that drives air hunger (Gandevia et al., 1993). Ward Fowler demonstrated that, at the break of a maximal BH, if the participant inhaled an anoxic gas mixture, the breath hold could be extended for a short period (Fowler, 1954). This indicated that, at least briefly, input from the peripheral and central chemoreceptors signalling gas exchange failure could be overridden by feedback from the pulmonary stretch receptors and chest wall.

It is also important to acknowledge that in addition to the brain's inherent vulnerability to BH‐induced hypoxia, given its obligatory high rate of O_2_ consumption in the face of limited glycolytic reserves (Scheinberg & Stead, 1949), it has evolved with heightened sensitivity to hypercapnia, reflecting prioritisation of acid–base balance for the stabilization of chemosensory and autonomic control at the level of the brainstem (Caldwell et al., 2021; Kety & Schmidt, 1948). The complexities of distinguishing between these competing albeit combinatorial vasoactive stimuli in the setting of BH physiology, while beyond the scope of the present review, has been considered elsewhere (Bailey et al., 2024, 2017; Bain et al., 2018).

To the present cases of human execution of convicted criminals and assisted suicide of a terminally ill patient with the ‘Sarco’ pod through inhalation of pure N_2_, the alveolar O_2_ concentration and $P_{O_{2}}$, which drives alveolar–blood O_2_ transfer, will be driven down at the rate of wash‐out of O_2_ as dictated by their lung volumes, respiratory rate, tidal volume and resultant alveolar ventilation as well as their extant metabolic rate (Poole & Bailey, 2024). Thus, within very few breaths, $P_{AO_{2}}$ and $P_{aO_{2}}$ will drop and, especially when $P_{aO_{2}}$ has fallen below 60 mmHg, the CBs will evoke a very powerful drive to breathe that is highly dyspnoeagenic. However, in contrast to breathing air with ∼21% O_2_, by increasing ventilation whilst breathing 100% N_2_, the $P_{aO_{2}}$ will plummet more precipitously and the CBs will be further stimulated, exacerbating dyspnoea with all its attendant fear and anxiety‐inducing sensations (Banzett et al., 2021; Ernsting, 1963; Iturriaga et al., 2021; West, 1995) (Figure 5a).

A particularly disturbing scenario comes to light considering the recent executions as outlined. The Stryker gurney, to which all convicted criminals were bound, has a chest strap that when tightened as intended will undoubtedly impair chest wall and corresponding lung expansion. In this situation, as the N_2_ inspirate stimulates the CBs to exert their powerful drive to breathe, evoking a pronounced elevation in motor drive to the inspiratory muscles, the desired lung expansion will not be possible, further compounding dyspnoea and air hunger. Eye witness reports from all executions attest to the prisoners undergoing a prolonged period – several minutes – of severe respiratory distress including gasping, choking, retching and moaning as they invariably fought against their restraints (Andone et al., 2024; Ortiz & Books, 2024).

Furthermore, and with the continued precipitous reduction in $P_{aO_{2}}$, the individual's level of consciousness would also be expected to rapidly deteriorate from clouded consciousness, to a confused state, delirium, lethargy, obtundation, stupor, hypersomnia, vegetative state, akinetic mutism and ultimately clinical brain death (Tindall, 1990). We have previously documented the lowest tolerable limits of arterial hypoxaemia and associated convective/diffusive components of the cerebral ‘O_2_ cascade’ that the healthy human can endure prior to LOC (Bailey, 2019a; Bailey et al., 2017). One of the studies cited involved normobaric (pure) N_2_ (hyper)ventilation, resulting in the lowest $P_{aO_{2}}$ recorded to date in the published literature (16 mmHg) and LOC within 17–20 s (Ernsting, 1963) that likely occurred within a predicted four to five breaths (Poole & Bailey, 2024). During the (infamous) Red Wing studies, non‐consenting psychiatric patients were subject to rapid inflation of a specialised cervical pressure cuff to induce cerebral ischaemia, albeit a different stimulus, for up to 100 s (Rossen et al., 1943). The LOC occurred more rapidly (7–8 s), given the absence of hyperpnoea or activation of the carotid sinus baroreceptor reflex; with continued ischaemia, convulsions, marked cyanosis, involuntary urination and defaecation, bradycardia, dilatation of the pupils and changes in reflexes were observed (Rossen et al., 1943).

A final caveat that we failed to highlight in our original critique (Macefield, 2024; Poole & Bailey, 2024) relates to the presumed and misinformed logic of state officials who contend that N_2_ asphyxiation in normobaria (i.e., N_2_ inspired at the prevailing barometric pressures encountered by inmates and patient in the ‘Sarco’ pod) will result in a ‘euphoric’ death. Perhaps these advocates are confused with the euphoric irrationality caused by nitrogen narcosis typically experienced by scuba divers, likely attributable to the anaesthetic effect of breathing N_2_ at high partial pressures (i.e., hyperbaria) (Dean et al., 2003). Also referred to as ‘rapture of the deep’ or the ‘Martini effect’, this is an entirely separate condition with concurrent neurological symptoms resembling those associated with alcohol, nitrous oxide (laughing gas), marijuana and benzodiazepine drug intake (Hobbs, 2008). While clearly not the focus of the present review, the molecular and cellular mechanisms responsible for nitrogen narcosis are widely debated (D'Agostino et al., 2009). The ‘lipid theory’ of narcosis/anaesthesia posits that an increase in membrane volume induces conformational changes in various membrane‐bound proteins that induce narcosis (Wlodarczyk et al., 2006). The competing ‘protein theory’ predicts N_2_ molecules bind to specific hydrophobic sites inside the postsynaptic ion channels of glutamate receptors (Trudell et al., 1998).

However, significant symptoms are only present when divers typically descend to 30 m (4 atmospheres) and beyond (Unsworth, 1966), which equates to a $P_{N_{2}}$ of 2374 mmHg (0.781 × 760 × 4), more than three times higher than that achieved by breathing pure N_2_ at 1 atmosphere ($P_{N_{2}}$ = 1.000 × 760 = 760 mmHg). Yet again, the simple physiological facts of science trump the speculative and self‐serving fallacy of the state.

## 7 CONCLUSION

The current review has explored the complex physiology of N_2_, an important gas that continues to fascinate and intrigue in equal measure. With N_2_ recently thrust into the public and scientific spotlight by the inhumane application of N_2_ gas asphyxiation for human execution of convicted criminals and assisted suicide in a terminally ill patient, we have presented the molecular bases underlying its Janus‐faced physiology. We hope we have applied our integrated physiological knowledge to help inform the controversial public debate and directly challenge the misconceived notion that N_2_ gas asphyxiation offers a humane death.

## AUTHOR CONTRIBUTIONS

Damian M. Bailey conceived the idea and wrote the first draft of the manuscript with input from Vaughan G. Macefield and David C. Poole. Damian M. Bailey, Vaughan G. Macefield and David C. Poole edited and revised the manuscript. Damian M. Bailey, Vaughan G. Macefield and David C. Poole approved the final version submitted for publication and agree to be accountable for all aspects of the work in ensuring that questions related to the accuracy or integrity of any part of the work are appropriately investigated and resolved. All persons designated as authors qualify for authorship, and all those who qualify for authorship are listed.

## CONFLICT OF INTEREST

D.M.B. is Editor‐in‐Chief of *Experimental Physiology*, Chair of the Life Sciences Working Group, member of the Human Spaceflight and Exploration Science Advisory Committee to the European Space Agency and member of the Space Exploration Advisory Committee to the UK and Swedish National Space Agencies. D.M.B. is also affiliated to Bexorg, Inc. (USA) focused on the technological development of novel biomarkers of cerebral bioenergetic function and structural damage in humans. V.G.M. is a Senior Editor and Opinions Editor of *The Journal of Physiology*. D.C.P. is Deputy Editor‐in‐Chief (USA) of *Experimental Physiology*. D.M.B., V.G.M. and D.C.P. were blinded from the review process and from making any editorial decisions for this manuscript.

## Data Availability

All data supporting the findings of the present study are available within the paper.
